# Age-Related Changes in Physical Fall Risk Factors: Results from a 3 Year Follow-up of Community Dwelling Older Adults in Tasmania, Australia

**DOI:** 10.3390/ijerph10115989

**Published:** 2013-11-11

**Authors:** Marie-Louise Bird, Jane K. Pittaway, Isobel Cuisick, Megan Rattray, Kiran D. K. Ahuja

**Affiliations:** School of Human Life Sciences, University of Tasmania, Locked Bag 1320, Launceston, Tasmania 7250, Australia; E-Mails: Jane.Pittaway@utas.edu.au (J.K.P.); ikcusick@postoffice.utas.edu.au (I.C.); mrattray@postoffice.utas.edu.au (M.R.); Kiran.Ahuja@utas.edu.au (K.D.K.A.)

**Keywords:** accidental falls, exercise, physical activity

## Abstract

As the population ages, fall rates are expected to increase, leading to a rise in accidental injury and injury-related deaths, and placing an escalating burden on health care systems. Sixty-nine independent community-dwelling adults (60–85 years, 18 males) had their leg strength, physical activity levels and their annual fall rate assessed at two timepoints over three years, (summer 2010 and summer 2013) monitoring balance. Force platform measures of medio-lateral sway range increased significantly under conditions of eyes open (mean difference MD 2.5 cm; 95% CI 2.2 to 2.8 cm) and eyes closed (MD 3.2 cm; 95% CI 2.8 to 3.6 cm), respectively (all *p* < 0.001) indicating worsening static balance control. Dynamic balance showed similar changes (*p* < 0.036). Leg strength was not significantly different between visits (*p* > 0.26). Physical activity reduced significantly (MD −909 Cal/week; 95% CI −347 to −1,470 Cal/week; *p* = 0.002) during the course of the study. Participants maintained aerobic activities, however resistance and balance exercise levels decreased non-significantly. The likelihood of falling was higher at the end of the study compared to the first timepoint (odds ratio 1.93, 95% CI 0.94 to 3.94; *p* = 0.07). Results of this study indicate that despite maintenance of leg strength there was an increase in medio-lateral sway over a relatively short time frame, with higher than expected increases in fall rates.

## 1. Introduction

As the population ages, fall rates are expected to increase, leading to a rise in accidental injury and injury-related deaths, affecting our society and placing an escalating burden on health care systems. The personal impact of falls is high, often resulting in loss of confidence and reduced functional activity, deconditioning and consequently an increased risk of falling [1]. Currently costing about 5% of the total health budget, it is expected that by 2051 fall related injuries will triple and will cost the Australian health sector over $ 1,375 million dollars per year [2].

There are many reasons why older people fall: some of these factors are environmental and others pertain to the tasks undertaken or the physical characteristics of the faller. These factors include balance impairment and reduced lower limb strength, both of which increase with natural aging processes. Older adults tend to move their centre of mass more (increased postural sway) when standing still, compared to younger adults. It is suggested that changes to postural sway control in older adults who fall may be due to a decrease in the sensitivity of their postural control system [3]. Lower limb weakness is a significant fall risk factor, with low muscle strength associated with an increase odds ratio of a fall of 1.76 (95% CI 1.31 to 2.37) and this value increases for the odds of falling multiple times if leg weakness is present 3.06 (95% CI 1.86 to 5.04) for recurrent falls [4]. The lower limb muscles involved include both ankle dorsiflexors [5] and knee extensors [6].

Many physical fall risks are potentially modifiable by lifestyle changes such as physical activity or exercise [7,8]. However, many older adults do not meet the current guidelines for physical activity for older adults, which include aerobic, resistance training and balance components. The American College of Sports Medicine (ASCM) recommends that older adults should partake of aerobic exercise, muscle strengthening exercises, and flexibility exercises [9]. In addition, individuals who are at risk for falling or mobility impairment should also perform specific exercises to improve balance. Specifically, aerobic exercise should consist of at least 150 min of moderate-intensity aerobic physical activity or at least 75 min vigorous intensity aerobic physical activity (or combination of these) throughout the week [9]. As well, strength training should be performed on at least two days a week, with balance exercise (or neuromotor training) of two hours per week also required to maintain health [9]. Age related declines in strength and balance can be addressed through targeted exercise [10,11].

Growing evidence indicates that adequate levels of physical activity are important in maintaining physical function and mobility, influencing balance, muscle and strength in older adults. Physical activity is defined as “any body movement that substantially increases energy expenditure” [12] and includes both occupational activities and leisure activities. An additional positive sequellae of adequate physical activity may be fall prevention [7]. Sedentary behaviour is associated with an increased incidence of falling (the odds of falling are increased 1.14 times), whereas increased physical activity in daily life yields significant reductions in falls that result in injuries [13]. 

The primary goal of this study was to determine changes in balance, leg strength, physical activity and previous 12 month fall rates at baseline and three years later in older independent living community dwelling adults. Our hypothesis was that there will be a decline in physical function and increase in fall rates between time points.

## 2. Methods

### 2.1. Participants

Men and women aged between 60 and 85 years of age were recruited for a study into seasonal variation in physical fall risk factors [14] including serum vitamin D levels [15], via local media (newspaper and radio) and local community clubs. The sample size calculation for the original study was based on a previous study reporting ML sway range in a sample of community-dwelling older adults (minimum effect size 2.5 mm sway; SD 8 mm; power 0.8, alpha 0.05) [16], and indicated n = 81 were required. Participants who completed this study were invited for reassessment three years after initial appointment.

All participants were community dwelling and physically independent. Exclusion criteria for the original study included: (1) if participants were currently suffering from, or had recently suffered from an acute medical condition; (2) if participants had an uncontrolled chronic condition or a history of stroke/other neurological disease; (3) if participants had a kidney or liver disease and/or (4) if participants were consuming greater than 800 IU of vitamin D daily. Furthermore, participants were withdrawn if they suffered a medical condition during the study period that affected their ability to perform the strength tests. This study was approved by the Human Research Ethics Committee (Tasmania, Australia) Network (HREC, reference number H0010561). Written, informed consent was obtained from all participants and they were free to leave the study at any time.

### 2.2. Testing Protocol

Static and dynamic balance, leg strength, physical activity and annual fall data were measured at two timepoints: at the end of summer 2010 and 2013. In the current study, assessment timepoints are referred to as the initial summer assessment (ISA), which occurred in 2010 and the final summer assessment (FSA), which occurred in 2013. 

### 2.3. Outcome Measures

#### 2.3.1. Static and Dynamic Balance

Medio-lateral sway ranges over 30 s were measured using a force platform (AMTI Accugait PJB 101, Watertown, MA, USA) for the conditions of eyes open (EO) and eyes closed (EC), with and without a underlying 6.5 cm piece of foam (FEO and FEC), using AMTI Balance Clinic software (version 1.4). Dynamic balance measures were the four square step test [17,18] and the timed up and go test [19].

#### 2.3.2. Leg Strength

Maximum isometric strength of the knee extensors and ankle dorsiflexors were measured using a spring based system that measured strength in kilograms [20]. The highest value maintained for 2–3 s from the three trials was recorded. Average ankle and knee strength was determined by averaging the left and right knee and ankle values.

#### 2.3.3. Physical Activity

The Community Healthy Activities Model Program for Seniors (CHAMPS) questionnaire was used to measure the cohorts physical activity levels at the ISA and the FSA [21]. This validated, graded questionnaire, measures the amount of time spent in particular activities over a typical week in the last month, enabling calculation of participants’ caloric expenditure (Cal/week). 

#### 2.3.4. Falls Diary and Recall

Each participant in the ISA received a printed calendar, on which they reported details of any falls and associated injuries prospectively A fall was defined as “an unexpected event in which the participant came to a rest on the ground, floor or lower level” [22]. A pre-paid envelope was provided for ease of return of the calendar. At the FSA, participants recalled the number of falls experienced in the previous 12 months. Data are reported as number of falls recorded for the relevant 12 months, the number of participants who fell at least once (number of fallers) during this time, and the number of participants who fell at least twice (number of multiple fallers) during this time.

### 2.4. Data Analysis

Data that were not normally distributed was treated to log transformation. Statistical analysis used mixed-methods linear regression corrected for repeated measures (STATA version SE12, StataCorp, College Station, TX, USA) to determine differences between ISA and FSA for all variables and to estimate the associations between balance and muscle strength and between physical activity and falls. Frequency data was compared using chi^2^. Logistic regression was utilized to compare fall data between ISA and FSA. Data from linear regression analysis is presented as mean difference and 95% confidence interval (CI), while that for logistic regression analysis is presented as odds ratio and 95% CI.

The caloric expenditure gained from the CHAMPS questionnaire was used to determine if participants met the ACSM guidelines for physical activity (>1,000 cal/week [9]). Participants were determined to have met the ACSM resistance exercise guidelines if they completed greater than or equal to 1 h/week on questions 37–39 in the CHAMPS questionnaire. Finally, participants were determined to have met the ACSM balance guidelines if they completed greater to or equal to 1 h/week on question 35 in the CHAMPS questionnaire. 

## 3. Results

Sixty-nine independently mobile community-dwelling adults agreed to be followed up three years after ISA [15] (60 to 85 years, mean 73.5 SD 6.9 years, 18 males and 51 females); all participants were of Western European descent, with stable chronic disease including hypertension, cardio-vascular disease, respiratory conditions and diabetes. They were followed up three years apart (summer 2010 and summer 2013) monitoring balance, leg strength, physical activity levels, and their annual fall data for the previous 12 months (Table 1). Reasons for non-attendance at FSA included ill health, travelling or declined to attend the appointment. Force platform measures of medio-lateral sway range increased significantly, indicating worsening balance control, between ISA and FSA under all conditions (eyes open and eyes closed and on foam insert) (Table 1). A *post-hoc* power calculation with a sample size of 69, based on the variable of medio-lateral sway from these results, confirmed that the study was adequately powered (Cohen’s d = 1.0). Dynamic balance reduced between these timepoints for both the four square test (*p* = 0.036) and the timed up and go test (*p* < 0.001). Leg strength was not significantly different between visits (*p* > 0.26). Physical activity reduced significantly (mean difference MD −909 Cal/week; 95% CI −347 to −1,470 Cal/week; *p* = 0.002) during the course of the study. The number of participants meeting the ACSM aerobic guidelines was the same at both timepoints (chi^2^ = 0, *p* = 1.0), whereas there was a decrease in the number of participants meeting resistance (chi^2^ = 0.58, *p* = 0.45) and balance (chi^2^ = 0.7, *p* = 0.40) guidelines, although this difference was not significant. 

**Table 1:** Physical function parameters of the cohort at ISA and FSA.

Variable *	ISA	FSA	Difference	95% CI	*p* Value
M-L sway (EO) (cm)	1.2 ± 0.6	3.7 ± 1.2	2.5	2.2 to 2.8	<0.001
M-L sway (EC) (cm)	1.4 ± 0.8	4.6 ± 1.5	3.2	2.8 to 3.6	<0.001
M-L sway (FEO) (cm)	2.7 ± 1.0	7.0 ± 4.2	4.3	3.3 to 5.2	<0.001
M-L sway (FEC) (cm)	4.9 ± 1.7	11.5 ± 6.2	6.6	5.3 to 7.9	<0.001
Four Square (sec)	8.6 ± 2.2	9.0 ± 2.5	0.4	0.0 to 0.8	0.036
TUG (sec)	6.8 ± 1.6	7.9 ± 2.2	1.1	0.7 to 1.4	<0.001
Ankle Strength (kg)	10.6 ± 4.3	10.7 ± 3.4	−0.1	−0.9 to 0.6	0.748
Knee Strength (kg)	19.5 ± 6.9	19.4 ± 6.9	−0.1	−0.9 to 0.7	0.818
Caloric Expenditure (Cal/Day)	4,337 ± 287	3,428 ± 278	−909	−348 to −1,471	0.002

* Data represented as mean ± SD. M-L = medio-lateral, EO = eyes open, EC = eyes closed, FEO = foam eyes open, FEC = foam eyes closed, TUG = Timed Up and Go, ISA = initial summer assessment, FSA = final summer assessment.

Fall rates increased over the time of the study with increases in the number of falls, number of people falling and number of people falling multiple times (Table 2). The likelihood of falling at FSA compared to ISA was odds ratio 1.93, 95% CI 0.94 to 3.94; *p* = 0.07, *i.e.*, almost twice the odds of falling. Associations between the postural sway variables and falls showed a significant relationship between fall number and postural sway under the conditions of eyes closed (*p* = 0.028) but not any other conditions of sway (eyes open or on the foam insert). As well, multiple fallers were also positively associated with postural sway with eyes closed only (*p* = 0.019).

**Table 2:** Fall rates, number of people falling at least once and number of people falling at least twice at ISA and FSA in previous 12 months.

Falls	ISA *	FSA *	chi ^2^	*p* Value
Number of falls	23	38	6.6	0.01
Number of fallers	15	24	2.9	0.09
Numbers of multiple fallers	9	14	1.3	0.25

* ISA=initial summer assessment, FSA=final summer assessment.

## 4. Discussion

Our hypothesis that there would be a decline in physical function and increase in falls rate has been partially confirmed, with declines in balance but not strength reported. Results of this study indicate that over relatively short time frame of three years, significant decrease in postural stability was seen. Despite maintenance of leg strength, there was also an increase in medio-lateral sway with higher than expected increases in fall rates. Only a minority of the participants at each assessment were meeting the current resistance and balance guidelines. 

All of the conditions of medio-lateral sway increased over the three year period of the current study. High rates of medio-lateral sway are associated with increased rates of falling and multiple falls, compared with individuals with better medio-lateral sway stability [23]. Research indicates that postural sway may be influenced by sensori-motor functioning [24], especially when the eyes are closed. Our research re-enforces this finding with a significant relationship between falls, multiple falls and postural sway with eyes closed. 

Both knee and ankle strength did not change over the duration of the current study. Physical activities that the participants completed may have allowed them to maintain strength in the knee extensors and ankle dorsiflexors. Previous research indicates that the relationship between leg strength and physical functioning is not linear [25]. For physical fall risks there may in fact be a threshold above which increases in strength do not have the effect of diminishing fall rates. Conversely, lower extremity strength values below threshold do have a relevant association to fall risk, [6]. Interestingly, postural sway appears to increase with increasing leg strength with the theory that weaker adults use co-contraction to limit their motion and thus provide stability [24].

Overall, a reduction in energy expenditure was seen between timepoints, but many participants (64 participants in both the ISA and FSA—93%) continued to meet the aerobic guidelines. Only a small percentage of participants met the resistance training guidelines at both timepoints, (21 participants in the ISA (30%) and 17 participants in the FSA (25%)), however this is more than previous research in the USA, where only 11% of older adults meet resistance training guidelines [26]. This may reflect either the current cohort and their choice of activities, or the impact of health promotion messages over the last twenty years. Even so, these levels of participation are still well below recommended guidelines [9].

Compliance with balance recommendations was the least met criteria with only four participants in the ISA (6%) and two participants in the FSA (3%) meeting the balance guidelines for older adults [9]. Activities recorded on the CHAMPS questionnaire like walking, gardening, housework, swimming and cycling are not specific or challenging enough to elicit changes in balance [27]. Information regarding participation in balance training and the proportion of older adults meeting the current recommendations does not appear to be well published. It would appear that promotion of current policies to increase balance and resistance training in older adult populations has not translated into practice. One explanation for this may be the habitual nature of exercise and the difficulties associated with lifestyle behaviour change for older adults. Novel approaches to behaviour change may be required. Recently, one such successful approach, the LiFE study [28], demonstrated the integration of requisite balance and strength activities into everyday tasks, for example, ironing or washing-up with a reduced base of support (balance) and squatting instead of bending (leg strength). The long term consequences of such interventions are still to be determined. Further research is needed in this area to translate current guidelines into practice.

Previous epidemiological data indicates that 30% of people in their sixties, 40% in the seventies and 50% in their eighties will fall each year [29,30]. The assumption with these statistics is that the overall increase in fall rates occurs in a linear fashion across the decades, with an average of 1% increase per year. Participants in the current study however, experienced a 6% increase in fall rates over 3 years. One explanation for this is that fall rates increase in a stepwise manner, rather than a linear fashion with threshold values for balance or physical activity being important contributors. We postulate that the lack of balance training may be a contributing factor to the increased fall rate seen, with the fall rate at the FSA being close to twice the rate at the ISA only three years earlier, however the magnitude of this contribution is difficult to quantify. This result may be a conservative estimate because of the potential underreporting at the FSA with use of the recall tool at that time rather than prospective data collection used at the ISA. Changes in function observed will be impacted on by both the aging process over the time of this study, as well as changes due to the falls themselves. Further research employing interventional studies with may test these hypotheses. 

The strength of following the same group of participants over time is that it allowed us to follow more closely, time-related trends in fall rates within individuals, compared to other studies, where cross-sectional studies which investigate different groups of people, at various times. Thus, the longitudinal design and the observational approach allow for ‘real-world’ assessment of the factors investigated in this study. The use of different tools to assess fall rate between ISA and FSA is a limitation of our study. We recognize the limitations of a recall tool for fall data collection, with a risk of under-reporting at the FSA.

## 5. Conclusions

This study shows an age-related decrease in postural stability over a short time-frame (three years) with implications for increases in fall rates, despite maintenance of leg strength. Low levels of participation in resistance training and balance training exist in this population of self-motivated healthy community-dwelling adults. This is despite current international guidelines for their inclusion for adults over the age of 65 years. Health promotion programs should invest more widely in disseminating current research findings.
